# Laser Nano-Neurosurgery from Gentle Manipulation to Nano-Incision of Neuronal Cells and Scaffolds: An Advanced Neurotechnology Tool

**DOI:** 10.3389/fnins.2016.00101

**Published:** 2016-03-11

**Authors:** Alessandro Soloperto, Gemma Palazzolo, Hanako Tsushima, Evelina Chieregatti, Massimo Vassalli, Francesco Difato

**Affiliations:** ^1^Neuroscience and Brain Technologies Department, Istituto Italiano di TecnologiaGenoa, Italy; ^2^Institute of Biophysics, National Research Council of ItalyGenoa, Italy

**Keywords:** laser nano-surgery, photo-polymerization, photo-stimulation, opto-transfection, intracellular surgery, laser dissection

## Abstract

Current optical approaches are progressing far beyond the scope of monitoring the structure and function of living matter, and they are becoming widely recognized as extremely precise, minimally-invasive, contact-free handling tools. Laser manipulation of living tissues, single cells, or even single-molecules is becoming a well-established methodology, thus founding the onset of new experimental paradigms and research fields. Indeed, a tightly focused pulsed laser source permits complex tasks such as developing engineered bioscaffolds, applying calibrated forces, transfecting, stimulating, or even ablating single cells with subcellular precision, and operating intracellular surgical protocols at the level of single organelles. In the present review, we report the state of the art of laser manipulation in neuroscience, to inspire future applications of light-assisted tools in nano-neurosurgery.

## Introduction

The etymology of the word “surgery” is derived from the ancient Greek word *cheirourgike*, or the Latin expression *chirurgiae*, which refer to the “hand work”. Conventionally, surgery is performed through the physical contact of tools operated by the hands of a highly skilled and experienced surgeon. Currently, manipulation of living matter has entered a new era exploiting alternative approaches and devices that assist or even substitute the human hands through tele-operated devices and miniaturized tools. As a prominent example, recent advances of laser technology and optical systems pushed the central role of light not only to observe the living matter at greater resolution, but also to perform tissue manipulation through “hands of light”. The resolution of the light touch is so precise that light represents, at present, the only tool providing access to real micro- and even nano-scale surgery.

Light manipulation tools can be classified in two main categories: gentle and invasive. The former non-invasive approach concerns the exploitation of optical forces, as in optical tweezers, to manipulate viruses, cells, as well as molecules in living cells (Oddershede, 2012). The latter consists of exploiting the energy of high photon flux to overcome the break-down threshold of the sample, and induce local ablation.

Despite the presence of a numerous literature proposing the adoption of optical trapping in surgical operations (Waleed et al., 2013), *in vivo* application of such an approach is still limited by the maximum forces that optical tweezers can generate, even with state of the art optical fiber-based set-ups (Liberale et al., 2007). Indeed, forces in the pico-newton range are certainly suitable for *in vitro* studies of single molecules and/or single living cells, but they are too small to apply a significant strain to induce local deformations or to move small entities in the crowded and dense environment of a living tissue. On the other hand, laser ablation is a much more effective tool applicable either *in vitro* or *in vivo*. Moreover, an accurate choice of the working parameters (laser wavelength, pulse energy, pulse duration, spatial beam profile, pulse repetition rate, and irradiation time) allows tuning the interaction of light with biological matter, from reversible manipulation to irreversible hard cuts (Rudhall et al., 2012).

Laser tools are highly controllable through dedicated electronics, which could be integrated in robotic surgery systems, enabling precise task definition and repeatability. Although research in laser surgery is still in its infancy for broad clinical applications, it has given an important contribution to understand the physiology (Hayes et al., 2012) of distinct pathologies at single cell level (Tilve et al., 2015), and it has demonstrated the potentiality to directly focus the area of intervention in living tissues with minimal scar formation. Indeed, applications of lasers in eye surgery or in laparoscopic systems are becoming widespread approaches in clinic (Mattos and Caldwell, 2012).

Moreover, laser technology opens the avenue for new applications in the emerging field of nano-medicine. Light can be used to trigger the action of chemically engineered nano-particles, designed to recognize molecular targets, and loaded with specialized photo-sensitizers. The action of light on such carriers can be multivalent: a caged compound can be delivered directly on the target tissue upon illumination (Yang et al., 2012), as well as a local enhancement of the light can induce a cell specific apoptotic effect (Pekkanen et al., 2014).

In the present review, prominent applications of laser nano-surgery will be highlighted, starting from the design of extracellular environment with micro- and nano-scale features, to the intracellular ablation of cellular compartments. All the reported examples, which are currently employed or could be exploited in the field of laser nano-neurosurgery, will provide an overview of the capability of laser manipulation.

## Laser processing of neuronal micro-environment: Engineering neuron-scaffold interactions

**Figure 1:**
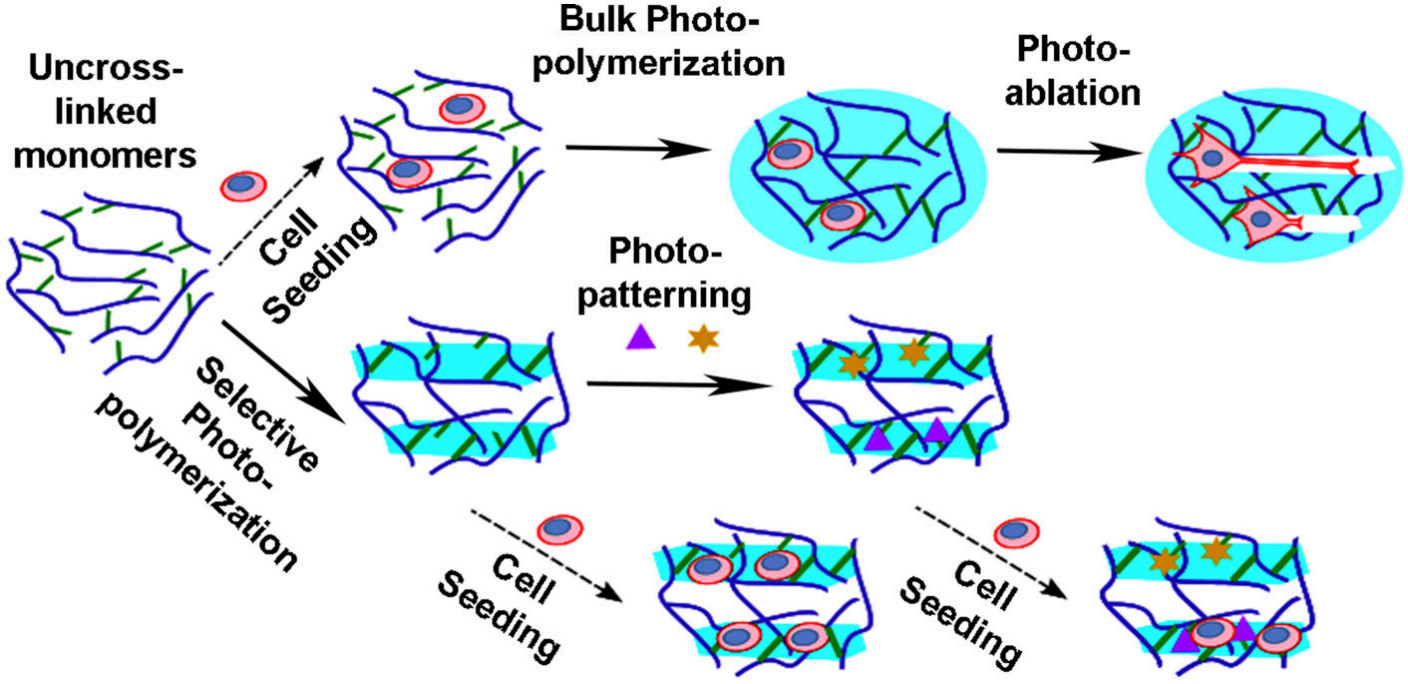
**Laser based approaches to produce bioscaffolds**. The photo-polymerization/ablation of polymers has permitted the production of complex polymeric structures which can be used as bioscaffolds. Hydrogels are produced from monomers (blue curves) by crosslinking of reactive groups (green lines). Neuronal cells can be seeded within or on the scaffolds prior or post-processing. Homogenous hydrogels (light blue circles) can be obtained by bulk photo-polymerization of the suspension. Then, physical channels (white rectangles) can be generated in the pre-formed gels by photo-ablation, in order to set out the path directing neurite elongation. Instead selected volumes (light blue rectangles) of the gel suspension can be photo-polymerized with the use of a focused laser or photo-masks. Biochemical properties can be further introduced by photo-patterning of peptides (purple triangles) and/or whole proteins (yellow stars), with function of grow factors, adhesion cues, enzymes, and so on.

Engineering of neuronal scaffolds is gaining importance to reproduce neural circuits *in vitro* as well as *in vivo* to repair injuries, to locally deliver cells or molecules, and to promote regeneration. Mimicking the extracellular physiological milieu remains a major challenge, because it is extremely heterogeneous in terms of topographical/mechanical/biochemical features. In this context, the use of light for modeling optically transparent three-dimensional (3D) hydrogels has been successfully applied to direct cell differentiation toward specific lineages or to promote and guide the outgrowth of neuronal processes. Design of topographical properties of such hydrogels can be accomplished by different strategies: photo-polymerization or photo-ablation (see Figure 1). Complex 3D structures can be photo-polymerized with a stereo-lithographic approach (Zorlutuna et al., 2011), or with ultrashort pulsed lasers exploiting the multi-photon absorption process within a femto-liter volume (Cumpston et al., 1999; Simitzi et al., 2015). Currently, super resolution techniques and development of new photo-resins are exploited to reduce the minimum size of features that can be generated (Scott et al., 2009; Gan et al., 2013). Indeed, there is growing evidence that the roughness of the surface could enhance cell differentiation, and direct the growth of neuronal projections (Bugnicourt et al., 2014; Marino et al., 2014). Otherwise, directional growth of neurons within a pre-existing hydrogel can be also obtained by post-processing of the scaffolds already containing the cells (Odawara et al., 2013). In this case, neurons are free to establish their hierarchical connectivity within the scaffold, and successive laser-mediated ablation of micro-channels opens the way for neurite sprouting (Sarig-Nadir et al., 2009). Otherwise, photo-cleavage of chemical moieties integrated in the hydrogel can locally modify hydrogel structures and allows the formation of *in vitro* neural networks with a specific geometry (McKinnon et al., 2014).

Mechanical properties of hydrogels can be also tuned by photo-degradation. Incorporating photo-sensitive moieties into the monomers that are assembled in the polymer backbone enables degradation of selected volumes by laser irradiation after the polymerization process. In particular, the spatial/temporal degradation of the hydrogel, leading to local modulation of the mechanical properties, can be finely controlled by the laser intensity (Kloxin et al., 2009), or by the use of a photo-mask (Lewis and Anseth, 2013). In this way the same scaffold could potentially mimic the mechanical properties of any tissue. Indeed, softening of a scaffold can be tuned to match the elastic properties of the nervous tissue to induce neural stem cell differentiation (Engler et al., 2006).

Biochemical functionalization of the extracellular environment through laser light can locally stimulate important cell functions. The first photo-chemical patterning of peptides in 3D hydrogels was performed by using photo-caged thiols, which upon irradiation with a focused laser, were freed to react with maleimide-modified peptides. The resulting biochemical channels promoted axonal sprouting (Luo and Shoichet, 2004). Further, photo-chemical immobilization of peptides or even whole proteins enables to pattern different growth factors in distinct volumes within the same 3D hydrogel (Wylie and Shoichet, 2011). Otherwise, two-photon laser scanning based lithography has been used in conjunction with photo-initiators to immobilize biomolecules during the photo-polymerization process at microscale resolution (Lee et al., 2008). Therefore, photo-patterning to generate biomolecular gradients (Owen et al., 2013), or concomitant functionalization with multiple proteins in 3D matrices (Wylie et al., 2011) allows achieving bioplatforms with high degree of complexity and mimicry of the native counterparts. Another possibility is to create hydrogels whose biochemical and physical properties can be reshaped by the cells themselves. For example, photo-polymerization of hydrogels containing enzymatically cleavable peptides enables the cell mediated remodeling of the scaffold by the expression of the cellular enzymes matrix metalloproteinases (Anderson et al., 2011).

Overall, the use of laser-based technologies within optically transparent biomaterials offers highly versatile tools for the processing and imaging of multifunctional scaffolding biosystems (Lewis and Anseth, 2013) that are suitable for neural tissue engineering.

## Gentle laser manipulation of neuronal cells

**Figure 2:**
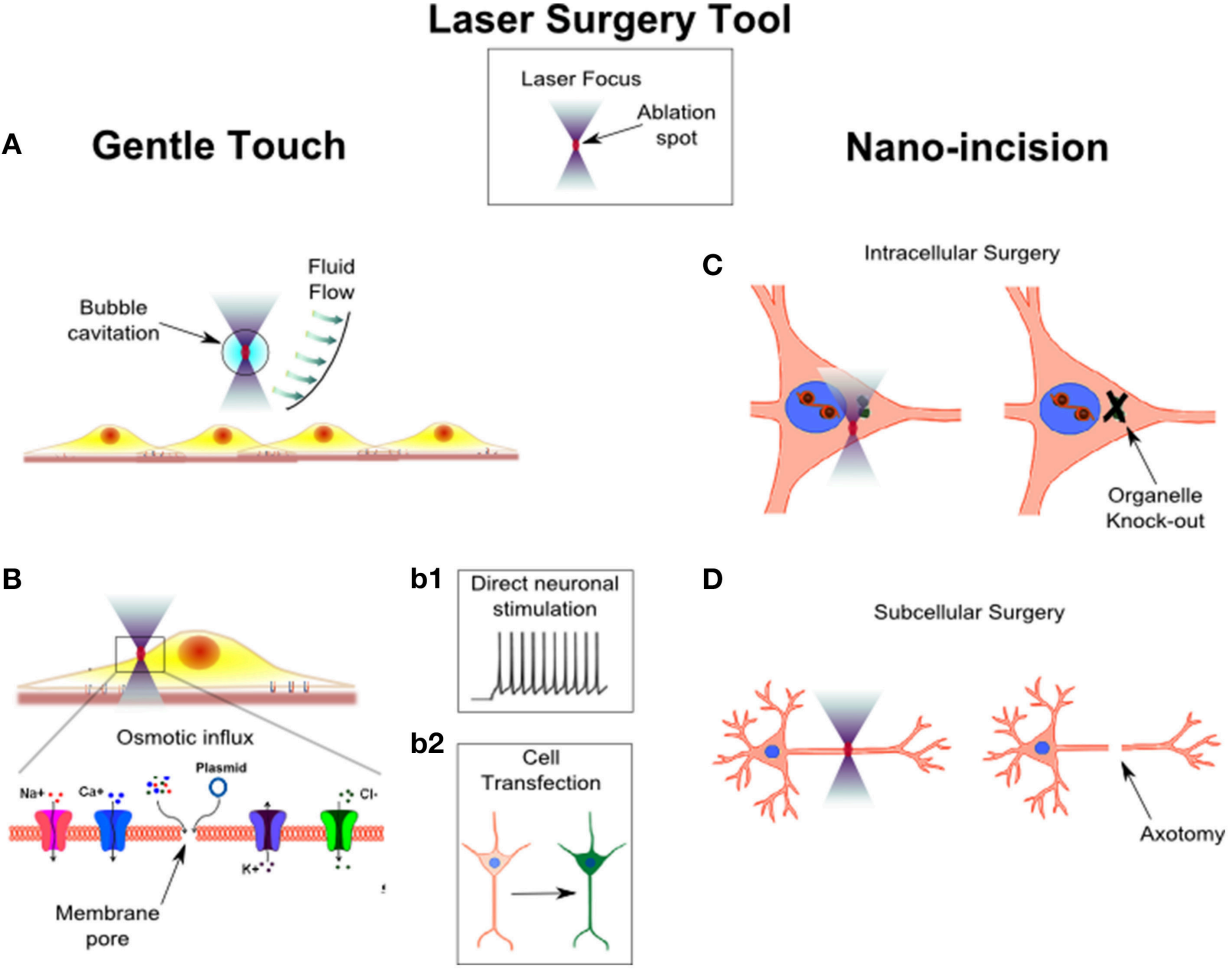
**Laser surgery tool: from gentle touch to nano-incision**. The surgery tool is based on a laser beam tightly focused in the sample. The ablation volume of the laser is restricted to a small volume in the center of the focal spot (red spot). An accurate choice of the laser type and a precise setting of laser power and number of delivered light pulse allow to modulate the invasiveness of the surgery tool from gentle touch to nano-incision. In panel **(A)**, the laser focus is not placed in direct contact with the cells, in order to induce a local micro-bubble cavitation. The micro-bubble induces a fluid flow which exerts shear stress forces on the nearby cells. In panel **(B)**, the laser focus is positioned on the plasma membrane of a cell. The laser generates a transient pore on the membrane (shown in the enlarged inset of the cell portion highlighted by the black box), through which an osmotic influx of solute occurs. In case of ions influx, the laser can induce a direct stimulation of a neuronal cell (**panel b1**). In case of plasmid influx, it is possible to transfect a cell which, for example, starts to produce a green fluorescent protein (**panel b2**). In panel **(C)**, the laser focus is positioned within the cell. Because the ablation occurs only in a small volume in the focus, the laser could be used to selectively disrupt an organelle of the cell. In panel **(D)**, the laser spot is scanned over a portion of the cell to produce a precise and confined cut, i.e., laser axotomy.

Probing and manipulating the complex structure and function of a neural circuit, without prompting any permanent morphological change, allows understanding the functional connectivity of neural circuits, and treating their pathological activities non-invasively. The interaction of light with living tissues induces focal perturbations (see the laser surgery tool in Figure 2), that can be transiently applied to perform cell stimulation. As an example, the possibility to locally generate shockwaves through microplasma-cavitation effects in water has been proposed as a high-throughput approach to exert mechanical stimulation of cells (Compton et al., 2014; Figure 2A). The ability to precisely control the mechanical environment of brain tissue is raising enormous attention especially after the discovery that the brain is one of the most mechanosensitive organs (Tyler, 2012). Inducing mechanical alterations would therefore allow to understand the mechanobiology of neuronal migration and development, providing important insights into the design of efficient neuronal scaffolds (Palazzolo et al., 2015). In other studies, a high-throughput mechanical stimulation of cells has been engaged to understand how abrupt stimulation of integrins could produce mild-traumatic brain injury (Hemphill et al., 2011; Grevesse et al., 2015).

Another way to achieve neuronal stimulation by laser irradiation is based on photo-thermal effects occurring in the laser focus. The earliest report of direct laser stimulation on neuronal cells was described by Fork on Aplysia ganglion neurons (Fork, 1971). The development of ultrashort pulsed sources incorporated in advanced two-photon microscopy set-ups permitted the exploitation of the non-linear excitation in the focal volume to directly stimulate neurons without using any caged compounds or fluorescent molecules (Hirase et al., 2002). Recently, microparticles or light sensitive conjugated polymers have been used to produce patterned photo-stimulation of neuronal circuits (Farah et al., 2013) or to develop new retinal prosthetic devices (Ghezzi et al., 2013). The main mechanism producing direct neuronal stimulation is photo-thermal but it does not require temperature-sensitive channels. Indeed, it was shown that thermal stimulation of cells was related to the rate of temperature increase (instead of the maximum temperature reached), which modified the capacitive properties of the cell membrane (Shapiro et al., 2012). However, depending on the light intensity delivered to the cells, other mechanisms could be involved in the direct stimulation of neurons (Beier et al., 2014), as seen in transient plasma membrane nano-poration (Figure 2Bb1). Although membrane opto-poration provides single cell stimulation, it cannot be exploited as an efficient protocol when repetitive cycles of stimulation are required. However, transient disruption of the cell membrane represents an intriguing approach to inject foreign molecules intracellularly, which in the specific case of nucleic acids injection, it can be used to achieve single cell opto-transfection (Figure 2b2). Tirlapur et al. (Tirlapur and König, 2002) reported the first evidence of optical-assisted delivery of foreign DNA into cells *in vitro*, thus showing the possibility to inject non membrane-permeable molecules. In such a way, it is possible to experimentally observe the consequences of delivering molecules into specific subcellular regions. For example, the introduction of specific mRNA in the dendrites or in the soma of living neurons highlighted the importance of subcellular localization of transcription factors in distinct cellular compartments (Barrett et al., 2006). A variety of lasers have been used to perform single cell opto-poration/transfection (Paterson et al., 2005; Marchington et al., 2010). Engineered beam shapes have been exploited to raise the throughput of opto-transfection (Rendall et al., 2012; Breunig et al., 2014). Recently, the application of extremely ultrashort femtosecond laser decreased the power necessary to nano-process cells (Uchugonova et al., 2008) by more than one order of magnitude. Therefore, optical re-programing of human cells into induced human pluripotent stem cells is becoming a safe and efficient approach (Breunig et al., 2015). Furthermore, opto-transfection of single neurons has been proposed in combination with optogenetic (Antkowiak et al., 2013), and technical efforts have been spent to apply the technique in an *in vivo* scenario (Ma et al., 2011). Optical transfection of single cells to induce expression of optogenetic probes could circumvent the need for viral particles or vectors to target specific cells, and it could provide a way to apply complex optical stimulation patterns with single cell resolution also on awake animals (Antkowiak et al., 2013).

## Surgery at the nano-scale: From intracellular ablation to subcellular dissection *in vivo*

Nano-fabrication has a tremendous impact in pharmaceutical and medical fields. Nano-scale knife (Kruskal et al., 2015), and implants are discovering new aspects in biology at the molecular scale (Betancourt and Brannon-Peppas, 2006). Proper control and manipulation of these nano-tools burden the development of robotic devices and automated control to operate them in living matter (Chang et al., 2010). On the contrary, the massless hands of light can be applied at the nano-scale through far field projection. Therefore, producing few nano-meters shift of the light focus requires physical displacement of active optical element in the micro-meter scale, which can be attained at several hundreds of kHz (i.e., using galvo mirrors, DMD, or AOD devices). Moreover, wavefront engineering enables the simultaneous projection of several light foci at distinct locations (Difato et al., 2012), or to modify the light wavefront to compensate the spherical aberration induced by the sample, in order to reach deep layers in tissues (Wang et al., 2014).

One of the most important aspects of laser surgery is the capability to overcome physical barriers, i.e., the cell membrane, with minimal perturbation in order to perform intracellular surgery in distinct compartments (Shen et al., 2005), such as the nucleus (König et al., 2001) or the cytoplasm (Colombelli et al., 2005). Laser irradiation has been applied to induce damage in submicron regions of the nucleus to study the molecular mechanisms underlying the repair of damaged DNA (Saquilabon Cruz et al., 2015). In addition, even though biotechnology offers several protocols to perform genetic engineering of cells and tissues (e.g., RNA*i*, gene knockouts, gene mutation), there is a growing demand for tools allowing intracellular manipulations at the level of single organelles. For example, intracellular laser surgery revealed that axonal elongation does not require a centrosomal microtubule organizing center (Stiess et al., 2010; Figure 2C).

Study on axonal differentiation and regeneration is another important application of laser nano-surgery (Figure 2D). Neurons are highly polarized cells which extend neurites that differentiate into several dendrites and a unique axon. When the axon is cut near the soma of the cell, one of the dendrites turns into a new axon. If instead the axotomy is performed far from the cell body, the proximal neurite stump tries to regenerate and regrow (Bradke et al., 2012). Several *in vitro* (Kim et al., 2009) and *in vivo* (Allegra Mascaro et al., 2013) models have been developed to evaluate the capability of different cell types to regenerate their injured axon. *In vitro*, a partial lesion of the axon can be induced with high repeatability, in order to study the age related ability of axonal regeneration, and to test various treatments to enhance axonal regeneration (Difato et al., 2011b). *In vivo* models of laser axotomy consented understanding whether axonal regrowth was correlated to a functional recovery (Yanik et al., 2004).

When laser nano-surgery is combined with monitoring devices associating a functional modification to the structural changes of neuronal circuits, it provides a powerful arena to develop repeatable injury models. Combining optical and/or electrical monitoring of neural networks with laser dissection *in vitro* (Difato et al., 2011a) produces simple and reliable injury models to test new prosthetic devices that restore the lost properties (Bonifazi et al., 2013). Indeed, laser nano-surgery facilitates to scale down the dimension of the injury to single connection, to single cell, to partial or complete lesion of an axonal bundle (Habibey et al., 2015), and thus test the efficiency of *in silico* neural network to recover distinct levels of lesion (Patel et al., 2012).

*In vivo*, laser nano-surgery can be employed to observe intrinsic homeostatic response restoring the synaptic density in local cortical circuits (Canty et al., 2013), to evaluate the detrimental effects of small strokes generated by laser induced clotting of microvessels (Nishimura et al., 2006), or to study the role of microglia when the vessels are completely disrupted (Davalos et al., 2005). Finally, laser scissors can be applied to precisely isolate cells within cultures, or subpopulation of cells from brain tissues to apply targeted proteomic studies (Drummond et al., 2015), to remove pathological tissues, e.g., brain tumors, or alleviate epileptic seizures (Medvid et al., 2015).

## Nano-composites assisted laser surgery

Nano-fabricated surgery tools are still challenging to handle, suffer mechanical vibration, and are fragile structures when inserted in tissues. On the contrary, light-based tools are contact free utensils which offer the advantages of avoiding contamination, and inducing negligible scars (Canty et al., 2013).

Moreover, the targeted delivery of caged moieties, photo-sensitizers, or the expression of light-sensitive ion channels provide an additional control on the nature of light-matter interaction. For example, genetic engineering of novel light sensitive constructs are now used to ablate specific cells *in vivo* or to dissect and inactivate specific proteins in living cells, thus achieving molecular surgery specificity (Williams et al., 2013).

Usually, surgical procedures have been classified in two types of interventions: the cutting aimed at the reshaping or removal of pathological tissues, and the manipulation and rejoining of healthy portion of a tissue. A better understanding of light-matter interaction could lead to not only perform surgery down to the molecular scale, but also to control and modulate the induced local effect. Therefore, we can assume that laser nano-surgery could establish a new surgical paradigm associated with a wider range of tissue manipulations: the choice of the targeted nano-composites and the light dose could produce either activation (Papagiakoumou et al., 2013)/sensitization (He et al., 2016), or inactivation/ablation of single cells as well as molecules in living tissues (Bergeron et al., 2015).

Finally, the development of compact and cost-effective diode-pumped lasers, which significantly reduce the complexity and price of multiphoton systems (König et al., 2015), together with the *in situ* enhancement of the light effect through engineered nano-compounds (Taratula et al., 2015), allows high resolution targeting deep in tissues, thereby paving the way for the future of laser nano-surgery in clinical applications.

## Author contributions

AS, GP, HT, EC, MV, and FD selected the literature and chose the topics of the review. AS, GP, MV, and FD wrote the manuscript and prepared the figure. FD supervised the study.

### Conflict of interest statement

The authors declare that the research was conducted in the absence of any commercial or financial relationships that could be construed as a potential conflict of interest.
